# A New Model of Repetitive Traumatic Brain Injury in Mice

**DOI:** 10.3389/fnins.2019.01417

**Published:** 2020-01-21

**Authors:** Kui Chen, Hao Gu, Liang Zhu, Dong-Fu Feng

**Affiliations:** ^1^Department of Neurosurgery, Shanghai Ninth People’s Hospital, Shanghai Jiao Tong University School of Medicine, Shanghai, China; ^2^Institute of Traumatic Medicine, Shanghai Jiao Tong University School of Medicine, Shanghai, China

**Keywords:** repetitive traumatic brain injury, animal model, diffuse axonal injury, inflammatory response, learning and memory

## Abstract

Repetitive traumatic brain injury (rTBI) is a major health care concern that causes substantial neurological impairment. To better understand rTBI, we introduced a new model of rTBI in mice induced by sudden rotation in the coronal plane combined with lateral translation delivered twice at an interval of 24 h. By routine histology, histological examination of Prussian blue-stained sections revealed the presence of microbleed in the corpus callosum and brain stem. Amyloid precursor protein (β-APP) and neurofilament heavy-chain (NF-200) immunohistochemistry demonstrated axonal injury following rTBI. Swelling, waving, and enlargement axons were observed in the corpus callosum and brain stem 24 h after injury by Bielschowsky staining. Ultrastructural studies by electron microscopy provided further insights into the existence and progression of axonal injury. rTBI led to widespread astrogliosis and microgliosis in white matter, as well as significantly increased levels of tumor necrosis factor (TNF)-α and interleukin (IL)-1β. rTBI mice showed a significantly increased loss of righting reflex (LRR) duration within each time point compared with that of sham animals, which was under 15 min. rTBI mice exhibited depression-like behavior at 1 month. rTBI mice also demonstrated deficits in MWM testing. These results suggested that this model might be suitable for investigating rTBI pathophysiology and evaluating preclinical candidate therapeutics.

## Introduction

Repetitive traumatic brain injury (rTBI) has been growing into a worldwide issue. In the United States, it is estimated that generally at least 5.50% of traumatic brain injury (TBI) patients experienced rTBI in the following year (Lasry et al., 2017). Compared with the general population, rTBI is more common in athletes of contact sports, including boxing and football (Mckee et al., 2009). In boxing, players experience repeated head hits during a single season (Greenwald et al., 2008), let alone throughout their whole boxing career. The situation is similar to that of football players, for whom the incidence of rTBI can be as high as 34.9% (Langburt et al., 2001). All these sports-related TBIs contribute to a cost of 60 billion dollars per year in the United States (Corso et al., 2006).

rTBI not only causes a heavy social–economic burden but also leads to serious public health problems. In 1928, Dr. Martland first described the clinical symptoms of rTBI in retired boxers. These symptoms included amnesia, cognitive disorders, and mental deterioration (Changa et al., 2018). Since it was first defined, growing attention has been given to clarifying the relationship between rTBI and its potential sequelae, especially cognitive dysfunctions and mental disorders. It seems that patients with rTBI are likely to have a poorer prognosis and are likely to suffer from severe and ongoing cognitive dysfunction, memory difficulty, and depression (Guskiewicz et al., 2003).

To better understand rTBI, different types of animal models have been established. Currently, popular experimental models are usually built on isolated linear impact injury (Dixon et al., 1987; Lighthall, 1988; Marmarou et al., 1994) or impulsive rotational accelerations (Xiao-Sheng et al., 2000; Namjoshi et al., 2014). However, in fact, nearly every injury process of rTBI is composed of both linear and rotational accelerations (Meaney and Smith, 2011; Karton et al., 2014), which means these models may not completely replicate the mechanistic injury process of rTBI. Meanwhile, it has been shown that there are diffuse axonal injuries (DAIs) in rTBI patients’ brains with sequelae (Geddes et al., 1999; Omalu et al., 2011). Herein, we proposed to introduce a new experimental model of rTBI using a modified simultaneous linear and angular head acceleration setup in mice. Furthermore, we performed pathological examinations and behavioral assessments in hopes of providing a better understanding for preclinical studies on rTBI.

## Materials and Methods

### Animals

Twelve-week-old (33.7 ± 4.8 g) C57BL/6 male mice purchased from Shanghai SLAC Laboratory Animal Co., Ltd., were housed in the animal facility of the Shanghai Ninth People’s Hospital affiliated with the Shanghai Jiao Tong University School of Medicine on a 12-h/12-h light/dark cycle with free access to standard food and water for 1 week before the experiments. All experiments were monitored by the Animal Care Committee of the Shanghai Jiao Tong University School of Medicine and were performed according to the guidelines of the Animal Care Committee of the Shanghai Jiao Tong University School of Medicine. All efforts were made to minimize the number of animals used and their suffering.

### rTBI Injury Model

**FIGURE 1:**
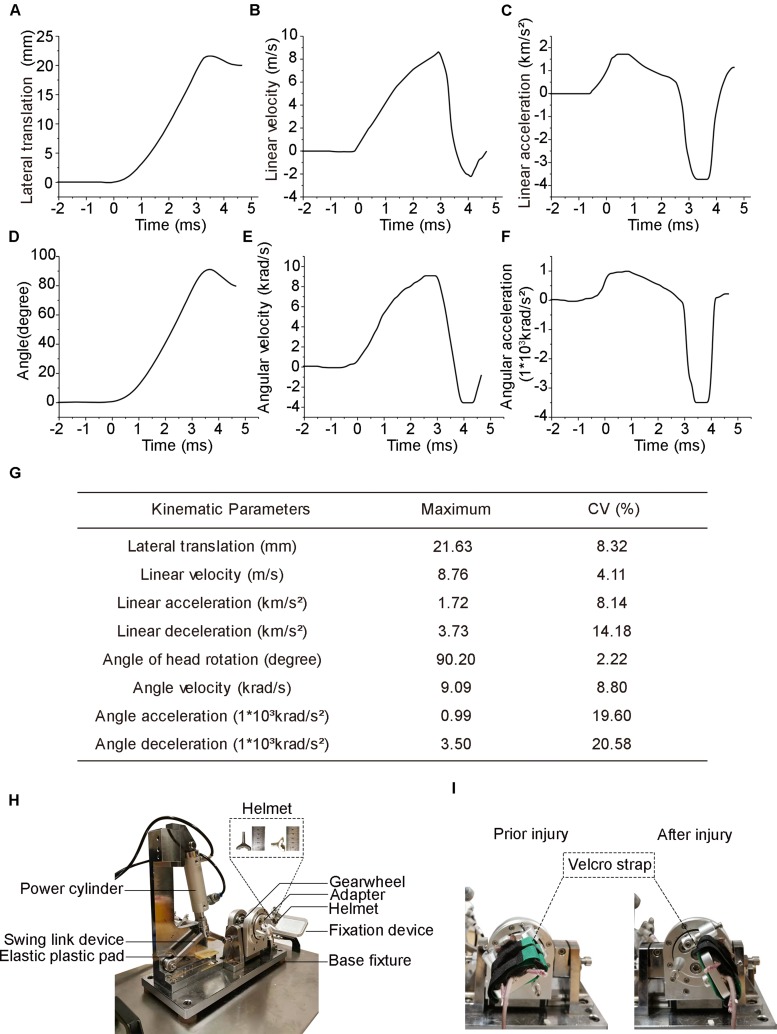
Head biomechanics of the injury device and actual photograph of the experimental setup. **(A)** Head lateral translation–time graph following injury. **(B)** Head linear velocity. **(C)** Head linear acceleration (positive value) and deceleration (negative value). **(D)** Head deflection is measured as the rotational angle of the coronal plane. **(E)** Head angle velocity. **(F)** Head angle acceleration positive value and deceleration negative value. **(G)** Summary of peak values of the head biomechanical parameters averaged across all eight mice. Data are presented as the means for each injury. **(H)** Photograph of the injury setup. **(I)** Photographs of the mice prior to and after injury.

Both sham-injury mice and rTBI mice were anesthetized with 5% isoflurane for 2 min in oxygen (1 L/min). However, the sham-injury mice were fixed onto the apparatus without starting the device. The experimental setup was designed according to our previous rat experimental animal model that consisted of a base fixture, a pneumatic cylinder and a motion form converter (Li et al., 2010). The converter included a slider, a precision guide, a pivot, and a gearwheel. The experimental setup was improved by removing the drawspring and introducing a tailored helmet matched well to the mouse head to ensure stability and a fixation device consisting of two Velcro straps and one concave titanium structures to fix the head and the body (Figure 1H). The helmet was designed according to the size of the mouse’s skull, which was made of titanium alloy with approximately 4-mm-thick and 15-mm inner diameter to facilitate consistent transmission of kinetic forces between the helmet and the head (Figure 1H). The fixation device was designed to induce rotation in the coronal plane combined with lateral translation to the mouse. The movements that happened to the mouse was in Figure 1I.

### Animal Model Preparation

The method for creating the experimental DAI model was used in this study according to our previous report (Li et al., 2010). Briefly, after induction of anesthesia, the head of the mice was secured to the head helmet. The body of the mice was positioned at the fixation device of concave titanium structure by the Velcro strap. For mice in the injury group, the angular displacement of the head and body was approximately 90.2 ± 2.0°, and the peak lateral was 21.63 ± 1.80 mm, whereas mice in the control group were freed from the device without exposure to acceleration loading.

The rTBI mice received two injuries 24 h apart. The investigators who performed behavioral and pathological tests, as well as data analyses, in this study were not aware of the study group assignments, which were revealed only after all analyses were completed.

### Biomechanical Measurements

The recording of heads’ motion trajectories in mice, the analysis of the video, and the evaluation of the rigidity of the head/helmet interface were performed as described previously (Li et al., 2010). Briefly, head motion was tracked with markers placed at the slider, the helmet, and the two lateral arms. These markers were tracked at 10,000 frames per second by a high-resolution digital imaging system (model HiSpec, FASTEC&IMAGING, United States). Coronal and lateral translational displacements of the helmet were calculated using motion analysis software (ProAnalyst 1.5, Xcitex Inc., United States). The linear displacement was converted to the rotational displacement of the helmet using the radius of the wheel gear. For the conversion, a scaling factor λ [λ = (mass of human brain/mass of mouse brain)^1/3^ = 13.8] (Namjoshi et al., 2014; Panzer et al., 2014) was used in the scaling of the animal data to the human head-equivalent kinematic parameters. Eight mice in the rTBI group were used for biomechanical measurements.

### Pathological Analysis

Mice were anesthetized with isoflurane and then sacrificed. After loss of the tail clamping reflex, mice were transcardially perfused with 0.9% saline (4°C) until the blood was flushed out, and internal fixation was performed by perfusion with 4% paraformaldehyde in 0.1 M phosphate buffer saline (PBS) (pH 7.4) for 15 min. Afterward, the brains were carefully and quickly removed. All brain tissues were fixed in perfusate for 24 h at 4°C and then dehydrated, defatted, and embedded with paraffin. After paraffin embedding, the cerebrum (−1.5 and −3.0 mm relative to bregma) was coronally sectioned while the cerebellum and brain stem (lateral 0.2–0.4 mm) were cut into sagittal slices (6 μm) and mounted on positively charged glass slides (Fisher, Superfrost Plus).

### Immunohistochemistry Staining

Sections for Prussian blue were stained by Iron Stain Kit (# HT20, Sigma-Aldrich, St. Louis, MO, United States), following the manufacturer’s instructions. Briefly, deparaffinized slides were then hydrated to deionized water, immersed in a freshly prepared solution of equal parts 5% potassium ferrocyanide and 5% hydrochloric acid for 10 min, then rinsed with deionized water, immersed in 2% pararosaniline solution for 5 min, then rinsed with deionized water, and rapidly dehydrated and covered.

Deparaffinized brain sections were incubated with 0.01 M sodium citrate (pH 6.5) at 100°C for 15 min for antigen retrieval, blocked with 3% hydrogen peroxide, and then incubated with amyloid precursor protein (β-APP, 1:200; Invitrogen Life Technologies) in 0.01 M PBS. Subsequently, the sections were incubated with a Sunpoly-HI Polymer Detection Kit (Sunpoly-HI, Shanghai Sunbiote, China) for 30 min at room temperature, followed by staining with the ABC Elite system with diaminobenzidine for visualization. Sections were counterstained with hematoxylin, dehydrated in graded alcohols, cleared in xylene, and cover-slipped. The procedures for neurofilament heavy-chain (NF-200), glial fibrillary acidic protein (GFAP), and ionized calcium binding adaptor molecule 1 (IBA1) immunohistochemical staining were the same as those for β-APP, except for the primary antibody solution (NF-200, 1:2,000, Sigma-Aldrich, AB5539; GFAP, 1:5,000; Abcam, ab33922; anti- IBA1, 1:5,000, Abcam, ab5076). A total of 12 animals contributed, three from the sham-injury group and nine from the rTBI group at 24, 72, 168 h post injury (three of each group post injury). Each slide was visualized with a bright-field microscope (Leica, Germany), and digital images were scanned without zoom and with a resolution of 1,920 × 2,448 pixels for further analysis.

### Immunohistochemical Quantification

Micrographs were obtained with an Olympus IX51 microscope (Olympus, Tokyo, Japan) fitted with a MicroImage video camera. For each animal, sets of coronal and sagittal (*n* = 3) sections were stained and analyzed by an observer blinded to experimental conditions using ImageJ software (US National Institutes of Health, Bethesda, MD, United States). Staining was defined by the hue–intensity–saturation option and then applied equally to all images. β-APP immunohistochemistry-stained neuronal cell bodies and NF-200-stained axons were quantified in the brain stem (lateral 0.2–0.4 mm). Three random micrographs at a magnification of × 400 in regions of interest (ROIs) were taken to obtain immunohistochemical parameters, primarily the mean integrated optical density (IOD). Three non-overlapping areas of 150 μm^2^ in the corpus callosum region and three non-overlapping areas of 200 μm^2^ in the brain stem were randomly selected within which the area of GFAP and IBA1 immunoreactivity was calculated and expressed as a percentage of the field of view.

### Immunofluorescence Staining

The immunofluorescence staining procedure was similar to that for immunohistochemistry staining, except that the sections were incubated with antibodies for tumor necrosis factor (TNF)-α (1: 500, Abcam, ab1793) or interleukin (IL)-1β (1: 200, Abcam, ab9722) at 4°C overnight and Alexa Fluor-555-conjugated F (ab′) or Alexa Fluor-488-conjugated F (ab′) 2 fragment goat anti-rabbit IgG (Life Technologies) at room temperature for 1 h. After washing, sections were mounted onto slides and covered with mounting medium containing DAPI (Vector Laboratories, Inc., H-1500). Immunofluorescence images were captured by confocal images (Leica SP8). A total of six animals contributed, three from the sham-injury group and three from the rTBI group at 24 h post injury.

### Bielschowsky Staining

Bielschowsky staining was performed as described previously (Adelson et al., 2001). In brief, tissue sections were deparaffinized and hydrated, then immersed in solution with 20% silver nitrate and capped for 20 min in the dark at 37°C, washed in distilled water, and then immersed in silver ammonia solution for 15 min. Next, sections were washed in ammonia water for 2 min and immersed in solution with 20% silver nitrate. Six milliliters of solution with 20% silver nitrate 20 ml, 95% alcohol 20 ml, and ammonia was then added, washed in ammonia water for 2 min, rinsed in distilled water, and fixed in 5% sodium thiosulfate for 2 min. Finally, the sections were washed with tap water, dehydrated, cleared, and fixed. A total of eight mice, four from the sham-injury group and four from the rTBI group at 24 h post injury, contributed.

### Transmission Electron Microscopy

For transmission electron microscopy (EM) examination, mice were perfused transcardially with 0.9% sodium chlorine (4°C) followed by 2.5% glutaraldehyde in 0.01 M PBS (4°C) at the time points described above. Samples of interest from the posterior corpus callosum, the hippocampus, and the brain stem were cut and trimmed into blocks of approximately 2 × 1 × 0.5 mm^3^ (length × width × thickness) and further postfixed with 3% EM grade glutaraldehyde. The tissues were then dehydrated and embedded in epoxy resin, and ultrathin sections were prepared for EM as described (Li et al., 2010). A total of 16 animals contributed, 4 from the sham-injury group and 12 from the rTBI group at 24, 72, and 168 h post injury (four of each group post injury).

### ELISA

For protein determination, half-brains were homogenized in RIPA lysis buffer. Endogenous TNF-α and IL-1β protein levels were quantified by commercial ELISA kits (Abcam, ab208348 and ab100704, respectively) following the manufacturer’s instructions. A total of 12 animals contributed, three from the sham-injury group and nine from the rTBI group at 24, 72, and 168 h post injury (three of each group post injury).

### Behavioral Assessment

#### Righting Reflex

The mice were placed in a supine position immediately after each injury, and the loss of righting reflex (LRR) was counted as the time interval from being placed in the supine position to the first sign of righting. A total of 20 mice, 10 from the sham-injury group and 10 from the TBI group, contributed.

#### Porsolt Forced Swim Test

The Porsolt forced swim test (FST) was performed 1 month after the second TBI application according to the method described by Porsolt et al. (1977). Mice were gently placed individually into glass cylindrical tanks (24-cm height × 12-cm diameter) filled with tap water to a height of 16 cm from the bottom (maintained at 23–25°C). The test length for mice was 6 min. The last 4 min of the 6-min testing period was used to record the total duration of immobility. Immobility refers to the passive floating of mice in the water or making only slight movements to stay above the water. Ten mice in the sham-injury group and 10 in the TBI group contributed to the righting reflex test and were then also used in the FST.

#### Tail Suspension Test

The tail suspension test (TST) was also performed 1 month after the second TBI application according to the method described by Steru et al. (1985). Mice were suspended 55 cm above the floor by an adhesive tape placed approximately 2 cm from the tips of their tails. Climbstoppers (4-cm length × 1-cm diameters, 1.5 g, transparent hollow polycarbonate tubing) were placed around the tails prior to applying the tape. Immobility time was recorded during a 6-min period. Immobility refers to the time the mouse hung passively or remained completely motionless. A total of 20 mice, 10 mice from the sham-injury and 10 from the rTBI group, contributed to the TST.

### Morris Water Maze

The Morris water maze (MWM) test was performed in the acute phase [acquisition trials on postinjury days (PIDs) 1–5 and probe trial on PID 6], the subacute phase (acquisition trials on PIDs 8–12 and probe trial on PID 13), and chronically at 1 month (acquisition trials on PIDs 31–35 and probe trial on PID 36) and 3 months (acquisition trials on PIDs 91–95 and probe trial on PID 96) after the second TBI administration. The maze was composed of a round black pool 120 cm in diameter and 50 cm in depth. A black platform 6 cm in diameter and 30 cm in height was placed in the northeast quadrant of the pool. The pool was filled with water at 22 ± 1°C, and the platform was hidden 1 cm under the surface of the water. In each trial, mice were released from one of the four directions (east, south, west, or north) and were allowed to swim. Each mouse was allowed 60 s to discover the underwater platform. When the mouse arrived at the platform, it was allowed to rest on the platform for 20 s. If the mouse did not find the platform within 60 s, it was guided to the platform and was allowed to remain there for 20 s. After each trial, the mouse was placed in a dry cage. Each mouse was tested across four trials starting from four different start positions each day for five consecutive days. On day 6, the probe trial was performed in 60 s, and the platform was removed for this trial. The amount of time the mice spent in each quadrant was recorded. Mouse movement was recorded using a video tracking system (DigBehv, Jiliang Software Technology Company, Shanghai, China), and the results (including latency and swimming distance) were collected and calculated for statistical analysis. The acquisition of the animal behavior data, as well as quantification, was performed under blinded conditions. A total of 87 mice, 40 in the sham-injury group and 47 in the rTBI group, contributed to the MWM test.

### Statistical Analysis

All data are presented as the mean ± SD. The data from LRR and MWM acquisition and probe trials were analyzed by two-way ANOVA followed by Sidak’s multiple-comparisons test. The FST and TST results and GFAP and IBA1 protein levels were analyzed by *t*-tests. The data for NF-200, β-APP, TNF-α, and IL-1β levels were analyzed with one-way ANOVA plus Dunnett’s multiple-comparisons test. All data statistical analyses were performed using GraphPad Prism (version 7.05, GraphPad Software Inc.). *P* < 0.05 indicated a statistically significant difference between parameters.

## Results

### Biomechanics of the Experimental Animal Model During rTBI

Head biomechanical parameters were obtained from videography using motion analysis software. The head reached a peak lateral translation displacement of 21.63 ± 1.80 mm (mean ± SD, same below) in 4.38 ± 0.12 ms (Figure 1A) and obtained a maximum linear velocity of 8.76 ± 0.36 m/s in 2.88 ± 0.08 ms (Figure 1B). The peak head linear acceleration was 1.72 ± 0.14 km/s^2^ at 0.57 ± 0.02 ms, while the peak linear deceleration was 3.73 ± 0.53 km/s^2^ at 3.26 ± 0.10 ms (Figure 1C). The mean duration of linear acceleration and deceleration was 2.89 ± 0.10 and 1.49 ± 0.07 ms, respectively (Figure 1C). The head experienced a peak rotation angle of 90.2° ± 2.0° within 4.38 ± 0.12 ms (Figure 1D) and reached its maximal angular velocity of 9.09 ± 0.81 krad/s at 2.52 ± 0.08 ms (Figure 1E). The peak angular acceleration was 990 ± 194 krad/s^2^ at 0.59 ± 0.02 ms, while the peak angular deceleration was 3,500 ± 720 krad/s^2^ at 3.28 ± 0.09 ms (Figure 1F). The mean duration of angular acceleration and deceleration was 2.89 ± 0.10 and 1.49 ± 0.07 ms, respectively (Figure 1F). A summary of the coefficient of variation in the main parameters was listed in the chart above (Figure 1G).

### Histopathological Changes in rTBI Mice

**FIGURE 2:**
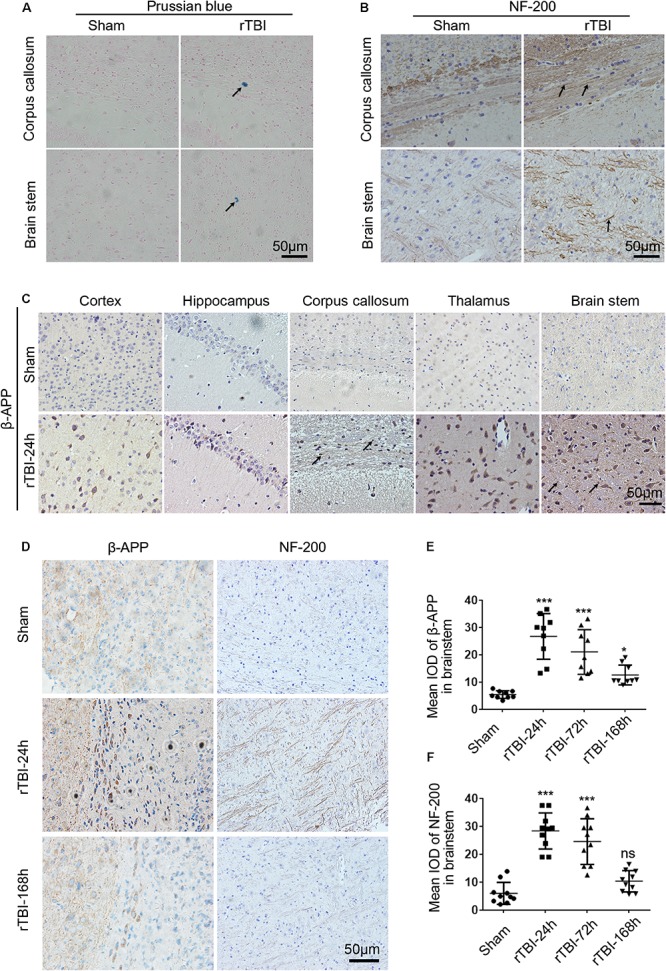
Histopathological changes induced by repetitive traumatic brain injury (rTBI). rTBI induced histopathological changes in Prussian blue, neurofilament heavy-chain (NF-200), and amyloid precursor protein (β-APP) in multiple brain regions. **(A)** Representative microbleed as identified by Prussian blue staining at 72 h post last injury; microbleed was indicated by a black arrow. **(B)** Immunochemical staining of mouse brain sections showing NF-200 staining in the corpus callosum and brain stem at 24 h after injury compared to sham-treated mice. Axons positively stained for NF-200 were shown with black arrows. **(C)** Immunochemical staining of mouse brain sections shows β-APP accumulation in neuronal cell bodies in the subcortical areas, hippocampus, corpus callosum, thalamus, and brain stem at 24 h after injury compared to sham-treated mice. β-APP immunoreactive staining of axonal profiles was observed as being granular in the corpus callosum and brain stem, as indicated by a black arrow. **(D)** Histopathology of β-APP and NF-200 staining in the brain stem white matter in mice following rTBI (24–168 h) compared to sham-treated mice. **(E)** The mean integrated optical density (IOD) of β-APP-stained neuronal cell bodies per micrograph as a function of time following rTBI in the brain stem. **(F)** The mean IOD of NF-200-stained axons per micrograph as a function of time following rTBI in the brain stem. ^∗∗∗^*p* < 0.001 and ^∗^*p* < 0.05; ns, no significance.

All injured mice survived without macroscopic evidence of a skull fracture or cerebral hemorrhage. However, cerebral microbleeds existed in the cortex, corpus callosum, hippocampus, and sagittal sections of the brain stem in injured mice at the sacrifice time of 72 h after last injury (Figure 2A). In the brains of the sham-treated mice, there were no similar identical expression (Figure 2A).

NF-200 and β-APP immunoreactivity were used to assess axonal injury in this model. In the sham group, scant NF-200-positive staining was observed, while many NF-200-positive staining axons were found in the corpus callosum and brain stem (Figure 2B). In sham-injured animals, β-APP immunoreactivity was not observed within nerve cell bodies in the brain at any sacrifice time (Figure 2C). However, 24 h after rTBI, there was a profound increase in β-APP immunoreactivity in neuronal cell bodies in the cortex, hippocampus, corpus callosum, thalamus, and brain stem (Figure 2C). β-APP-positive axonal profiles were also found in the corpus callosum and brain stem, and these immunoreactive axonal profiles were observed as being granular (Figure 2C).

Next, β-APP immunoreactivity in neuronal cell bodies and NF-200-positive staining axons were found to change over time in the brain stem (Figures 2D–F). As shown in Figures 2D,E, β-APP in neuronal cell bodies was significantly increased at 24 h post last injury, and the expression level was still significantly increased at 72–168 h. No NF-200-positive axonal profiles were observed in sham-injured animals, but at 24 h post injury, numerous axons with intense NF-200 staining were found in the brain stem (Figures 2D,F). However, at 168 h post injury, compared with acute time points, there was no difference in NF-200-positive staining between the sham and injury groups (Figures 2D,F).

### Bielschowsky Staining and EM Analysis of Traumatic Axonal Injury After rTBI

**FIGURE 3:**
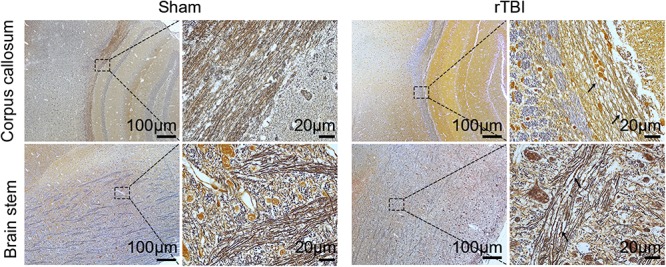
Post-repetitive traumatic brain injury (rTBI) axonal pathology was assessed by Bielschowsky silver staining. Representative immunohistochemical images showing the corpus callosum and brain stem at 24 h after last traumatic brain injury (TBI). The swelling, waving, and enlargement axons were observed (arrow indicated).

To detect post-rTBI axonal damage, Bielschowsky silver staining was used, which was a marker of axonal integrity (Figure 3). In comparison with the results in control mice, rTBI-induced loss of axons was evident in the corpus callosum and brain stem. The swelling, waving, and enlargement axons were also observed in the rTBI group in the corpus callosum and brain stem at 24 h post trauma.

**FIGURE 4:**
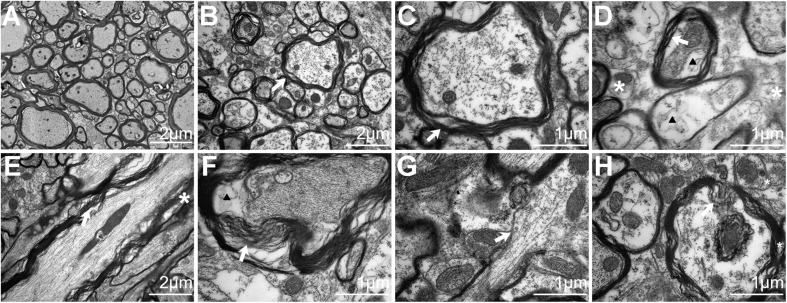
Electron microscopy analysis of traumatic axonal injury after repetitive traumatic brain injury (rTBI) in the brain stem. **(A)** Axons were consistently intact and organized in sham-treated mice, and mitochondria were normal in the brain. **(B)** A transverse section shows dystrophic myelinated axons at 24 h post injury. Focal separation of the axolemma and myelin sheaths was also observed (white arrow). **(C)** High-magnification cross-sectional view of **(B)** showed dystrophic myelinated axons and myelin sheath delamination (white arrow). **(D)** High-magnification cross-sectional view of injured axons; notable myelin sheath delamination (white arrow) was observed at 24 h post trauma. Focal intracellular edema (indicated by an arrowhead) and swollen mitochondria (white asterisk) were also present. **(E)** A longitudinal section through an injured axon (white arrow) showed more myelin sheath delamination and more cytoskeletal filaments (white asterisk) disrupted at 72 h post injury. **(F)** Transverse section showed axons with partial axoplasmic collapse (white arrow) and intracellular edema (arrowhead) at 72 h post trauma. **(G,H)** At 168 h after injury, advanced stages of axonal damage were characterized by small regions of focal myelin loss (white arrow) and intra-axoplasmic abnormal electron-dense material (white asterisk) with a longitudinal section and a transverse section, respectively.

The presence of axonal injury in rTBI was further confirmed by electron micrographs and provided further insight into the pathological progression from 24 to 168 h post trauma in the brain stem (Figure 4). In the control group, the axons and myelin appeared intact and organized consistently (Figure 4A). At 24 h, EM showed dystrophic myelinated axons and myelin sheath delamination (Figures 4B–D) and swollen mitochondria (Figure 4D). At 72 h, more myelin sheath delamination and more disrupted cytoskeletal filaments were observed (Figures 4E,F). At 168 h, small regions of focal myelin loss were found in the brain (Figure 4G), and intra-axoplasmic abnormal electron-dense material was also present in the brain (Figure 4H).

### rTBI Induced Widespread Astrogliosis, Microgliosis, and Increased TNF-α and IL-1β Levels

**FIGURE 5:**
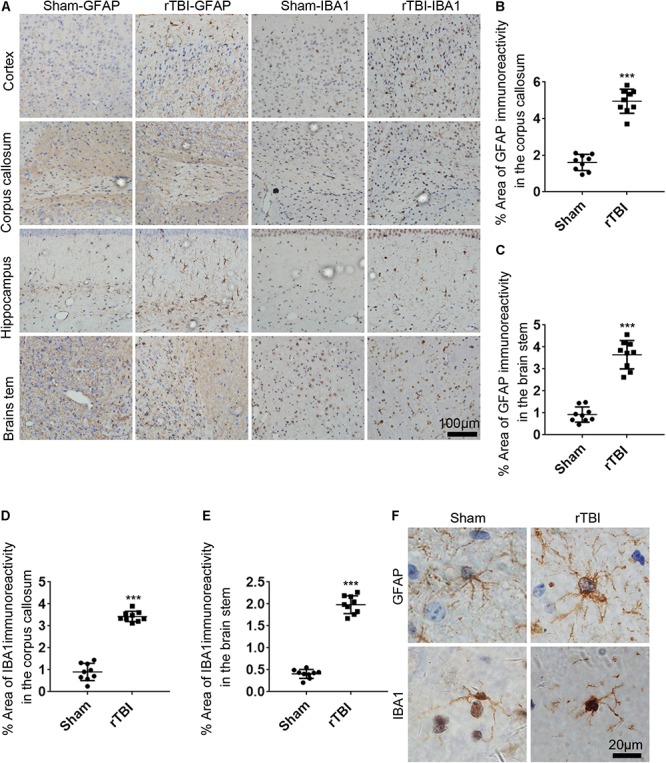
Repetitive traumatic brain injury (rTBI) induces widespread astrogliosis and microglial activation. **(A)** glial fibrillary acidic protein (GFAP) and ionized calcium binding adaptor molecule 1 (IBA1) staining in the cortex, corpus callosum, hippocampus, and brain stem 72 h after rTBI or the sham-treated group. **(B)** Quantitative analysis of the intensity of GFAP staining in the corpus callosum. **(C)** Quantitative analysis of the intensity of GFAP staining in the brain stem. **(D)** Quantitative analysis of the intensity of IBA1 staining in the corpus callosum. **(E)** Quantitative analysis of the intensity of IBA1 staining in the brain stem. **(F)** Representative × 100 magnified images showing the morphology of GFAP-stained astrogliosis and IBA1-stained microglia in the hippocampus. Data are presented as the mean ± SD (*t*-test, ^∗∗∗^*P* < 0.0001).

To determine the astrogliosis response and microglial activation after rTBI, GFAP immunostaining and IBA1 immunostaining were performed. For mice subjected to rTBI, immunostaining for GFAP revealed evidence of reactive astrogliosis in regions of the cortex, the corpus callosum, the hippocampus, and the brain stem at 3 days post injury (Figure 5A). In the corpus callosum and brain stem, rTBI (Figures 5B,C) groups showed a significant increase in GFAP immunoreactivity compared with that in their respective sham controls (*t*-test, *P* < 0.0001). We observed significantly increased microglial activation throughout several white matter tracts, including in the cortex, the corpus callosum, the hippocampus, and the brain stem of injured brains, compared with that in the sham controls, as assessed by microglial density (Figure 5A). In the corpus callosum and brain stem, rTBI (Figures 5D,E) groups also showed a notable increase in IBA1 immunoreactivity compared with that in their respective sham controls (*t*-test, *p* < 0.0001). A high-magnification view of the rTBI brain revealed that some GFAP-immunostained astrocytes displayed hypertrophic changes, while the immunodetection of GFAP revealed astrocytes in an unchallenged state in the sham brain (Figure 5F). The microglial morphology revealed that microglia in sham animals displayed highly ramified and extensively branched processes characteristic of the resting state (Figure 5F). In contrast, microglia in the corpus callosum, the hippocampus, and the brain stem of injured animals become predominantly hypertrophic to bushy morphology with primary branches (Figure 5F).

**FIGURE 6:**
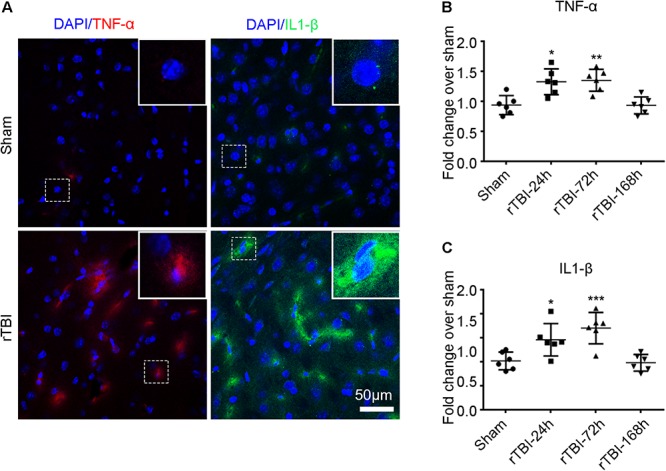
Repetitive traumatic brain injury (rTBI) increased proinflammatory cytokine levels. **(A)** Representative tumor necrosis factor (TNF)-α and interleukin (IL)-1β immunostaining for inflammatory cytokines in the brain stem between sham and rTBI groups at 24 h post injury. Bar graphs represent the mean ± SD percentage fold change in TNF-α **(B)** and IL-1β **(C)** levels in rTBI brain lysates compared to the levels in the sham brain lysates at the respective time points. For both graphs, ^∗^*P* < 0.05, ^∗∗^*P* < 0.01, and ^∗∗∗^*P* < 0.001 comparing rTBI versus sham, using one-way ANOVA and multiple *t*-tests with Dunnett’s corrections for multiple comparisons.

In addition to the astroglial response and microglial activation, we found that TNF-α (red) and IL-1β (green) immunostaining levels were significantly higher at 24 h post injury in the brain stem by immunofluorescence technique than levels in the sham group (Figure 6A). The levels of TNF-α (Figure 6B) and IL-1β (Figure 6C) were significantly higher at 24 and 168 h post TBI than the sham levels (one-way ANOVA plus Dunnett’s multiple-comparisons test, *P* < 0.05).

### Behavioral Deficits Induced by rTBI

**FIGURE 7:**
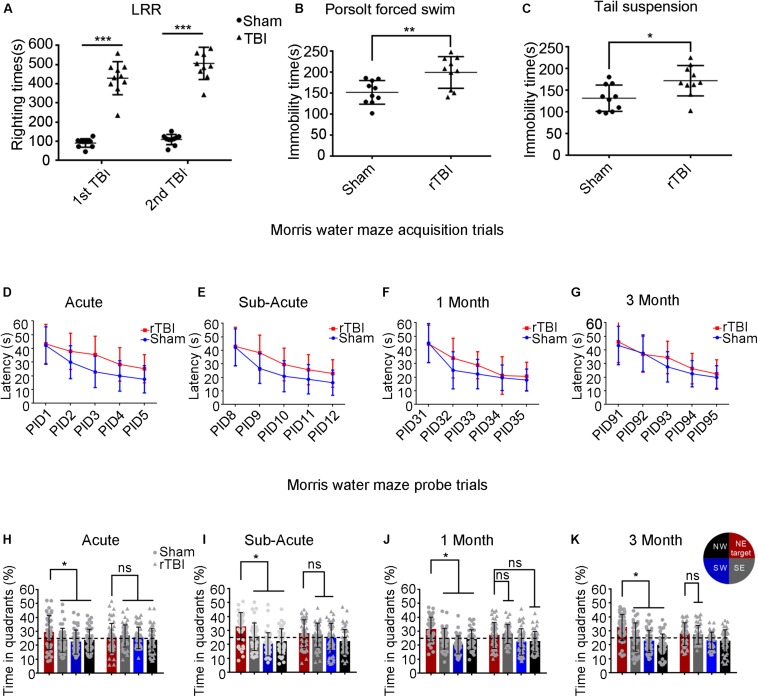
Repetitive traumatic brain injury (rTBI) results in behavioral deficits. **(A)** The duration of loss of righting reflex in rTBI mice was longer than that in sham-treated mice after every instance of traumatic brain injury (TBI); two-way ANOVA, Sidak’s multiple-comparisons test, ^∗∗∗^*P* < 0.0001 versus sham. **(B,C)** At 1 month post injury, rTBI mice demonstrated increased immobility time in the Porsolt forced swim test and tail suspension test; *t*-test, ^∗∗^*P* < 0.01, ^∗^*P* < 0.05 versus sham. **(D–K)** Results from the Morris water maze learning trials acquisition **(D–G)** and memory probe trial **(H–K)**; the dot represents the sham group, and the triangle represents the rTBI group. ^∗^*P* < 0.05, ns, not significant; *P* > 0.05. Values are mean ± SD. NE, northeast (target); NW, northwest; SE, southeast; SW, southwest.

To assess the severity of TBI, we assessed the duration of LRR. Mice subjected to rTBI in this model showed significantly increased LRR duration within each time point compared with sham animals [Figure 7A; TBI effect: *F*(1,36) = 279.3, *P* < 0.0001].

To determine whether injured mice exhibited depression-like behavior, we tested immobility time in the Porsolt FST and the TST at 1 month postinjury (Figures 7B,C). As shown in Figure 7B, in the Porsolt FST, mice subjected to rTBI showed significantly increased immobility time compared with that in sham animals (*t*-test, *p* = 0.0053). Similar analyses were carried out on data from the TST (Figure 7C), which suggested that immobility times differed significantly between the injury and sham groups (*t*-test, *p* = 0.0131).

To detect whether mice subjected to rTBI manifested spatial learning and long-term memory deficits, we subjected the mice to the fixed-platform version of the MWM. Both the rTBI and sham mice showed daily improvements in locating the hidden platform during the acquisition trials; however, acquisition deficits were observed in rTBI mice. At the acute and subacute time points, two-way ANOVA with Sidak’s multiple-comparisons test for acquisition trials shows that the main effect of time, the main effect of injury group, and the interaction between time and injury in the rTBI and sham groups were significant [acute: *P*_time_ < 0.0001, *F*(4,430) = 41.24; *P*_injury_ < 0.0001, *F*(1,430) = 40.47; *P*_time * injury_ = 0.0479, *F*(4,430) = 2.419; two-way ANOVA; subacute: *P*_time_ < 0.0001, *F*(4,430) = 53.60; *P*_injury_ < 0.0001, *F*(1,430) = 37.79; *P*_time * injury_ = 0.0463, *F*(4,430) = 2.441; Figures 7D,E]. At 1 month post trauma, the main effect of time and the main effect of injury group were still statistically significant, but the interaction between time and injury groups did not reach significance [*P*_time_ < 0.0001, *F*(4,430) = 62.64; *P*_injury_ = 0.0010, *F*(1,430) = 10.97; *P*_time * injury_ = 0.0726, *F*(4,430) = 2.161; two-way ANOVA; Figure 7F]. At 3 months post trauma, the main effect of time and the main effect of injury group were also statistically significant, but there was no significant interaction [*P*_time_ < 0.0001, *F*(4,410) = 52.30; *P*_injury_ = 0.0106, *F*(1,410) = 6.593; *P*_time * injury_ = 0.3576, *F*(4,410) = 1.0973; two-way ANOVA; Figure 7G].

At the acute time point, we found that differences between sham and rTBI mice were statistically significant at PIDs 2–5 (Figure 7D). At the subacute time point, the analyses found statistically significant differences between the control and rTBI groups at PIDs 9–12 (Figure 7E). At 1 month post injury, we found statistically significant differences between the two groups at PID 32 (Figure 7F). At 3 months, only differences at PID 93 were found to be significant in multiple comparisons (Figure 7G).

During probe trial testing, the differences in the percentage of time spent in the quadrants were first analyzed to evaluate the preference for the target quadrant versus the other three quadrants. We found that mice from the control group spent significantly more time in the target quadrant, which had contained the platform during training, than in the other three quadrants at the acute, subacute, 1-month, and 3-month time points post injury (*P*_acute_ < 0.05; *P*_subacute_ < 0.05; *P*1_month_ < 0.05; *P*3_month_ < 0.05; Sidak’s multiple-comparisons test) (Figures 7H–K). However, rTBI mice showed spatial memory impairment, as indicated by failing to show significant differences in time spent in the target quadrant compared with the other quadrants (Figures 7H–K). We did not find that mice in the rTBI group spent significantly more time in the target quadrant than in any other quadrants at the acute time point (*P* > 0.1; Sidak’s multiple-comparisons test). The rTBI group did not show a preference for the target quadrant compared with the other quadrants at subacute, 1-month, and 3-month time points post injury.

## Discussion

The major objective of this study was to introduce a new mouse model induced by simultaneous linear and angular acceleration to the head that replicates fundamental aspects of human mild rTBI. The parameters of this model were comparable to human mild rTBI. These combined accelerations in mice resulted in β-APP immunoreactivity, axonal injury, neuroinflammation, depression-like behaviors, and behavioral and memory deficits. This model may provide us with a new way to understand rTBI.

### Model Analysis

Currently, fluid percussion injury (FPI) models (Dixon et al., 1987), controlled cortical impact (CCI) models (Lighthall, 1988; Dixon et al., 1991), weight drop injury (WD) models (Marmarou et al., 1994), blast injury models (Cernak et al., 1996), and closed head injury models (Shitaka et al., 2011; Namjoshi et al., 2014; Sauerbeck et al., 2018) are the most common models used to induce TBI. FPI models are highly reproducible and can subtly adjust injury severity, but the craniotomy performed in preparation and high mortality weaken its comparability to rTBI (Dixon et al., 1987; Mcintosh et al., 1989). CCI models take advantage of high reproducibility and low mortality (Lighthall, 1988; Dixon et al., 1991). However, the need for craniotomy and the obvious tissue destruction compromises CCI similarity to rTBI, as most rTBIs in daily life usually occur without skull fracture (Namjoshi et al., 2014). Although the biomechanics of the WD model was similar to those of human TBI, the variability of uncontrollable factors, such as friction and air resistance in the guide tube, restricts its usage for rTBI study (Marmarou et al., 1994). Blast injury models are specifically designed for human explosive injury; sonic waves accompanied by blasts are transmitted rapidly in brain tissue in a short time, causing damage (Cernak et al., 1996; Goldstein et al., 2012). The biomechanics of blast injury models in mice have suggested that this injury pattern is similar to that of multiple severe concussion injuries in milliseconds, leading to apparent skull fractures (Xiong et al., 2013). The unique biomechanics and serious injury severity of blast injury models make them unsuitable for the research of rTBI in daily life. Recently, a Closed-Head Impact Model of Engineered Rotational Acceleration (CHIMERA), which defines energy to a closed skull with unconstrained head motion after impact characterized by impact as well as linear and rotational acceleration, and a tunable, monitored model of murine non-surgical, diffuse closed-head injury—modCHIMERA—which is based on the CHIMERA platform (Namjoshi et al., 2014; Sauerbeck et al., 2018), were introduced. These two modes replicate the fundamental aspects of human impact TBI by accurately delivering known biomechanical inputs very well. It is worth noting that in these models, the sagittal head motion without body movement decreases its resemblance to the majority of human mild TBI, as most human TBI such as those incurred in the National Football League (NFL), American football, and bicycling, occur when the body and head move together at high speeds and the head suddenly suffers a rapid change in head velocity.

In this study, the new rTBI model induced TBI obviously without mortality, which also inherited the main advantages of the DAI rat model (Li et al., 2010), including combined acceleration injury mechanisms, the convenience associated with being surgery free, and the consistency and adjustability of kinetic force provision. However, the new rTBI model did not replicate the injury process twice. To reduce injury severity, the drawspring used for power was removed, and a tailored helmet matched well to the mouse head ensuring stability and a fixation device, which was used to protect the cervical cord by using a Velcro strap to fix the mouse on a square silicone rubber pad and titanium plate to rotate the body and the head together, were introduced.

The time interval of rTBI has been deeply investigated, and previous studies have indicated that there is a significant period of vulnerability between two instances of TBI. One study reported that a second injury occurring within 3–5 days after the first injury caused more serious and lasting axonal injury and behavioral defects; however, no obvious cognitive defects were observed in the mice who experienced a second injury 7 days following the first injury (Longhi et al., 2005). Another study further found that pathological injury caused by a 24-h interval was more serious than that caused by a 48-h interval (Bolton and Saatman, 2014). These results indicated the existence of transient vulnerability in the brain from within 24 h of impact to 5 days after an initial impact. Thus, to fully understand the cumulative effects of rTBI, repeated injury was performed on mice 24 h after the first injury in this study.

### Biomechanical Analysis

A high-speed video was used to assess the biomechanical properties of the TBI model, which enabled the biomechanical parameters of the mouse head kinematics to be scaled to humans. As equal stress/equal velocity is the most commonly used technique to scale impact conditions between humans and animals (Gutierrez et al., 2001; Viano et al., 2009), the velocity of mice thus was equivalent to that of humans. The peak linear velocity of this device was 8.76 m/s, which is higher than average impact conditions (7.2 m/s) that cause concussion in the NFL but lower than elite impact conditions (9.0 m/s). In addition to velocity, the mouse data with the device were converted to their equivalent human values with a scaling factor λ [λ = (human brain mass/mouse brain mass)^1/3^ = 13.8], which has been widely used in the study of impact biomechanics (Gutierrez et al., 2001; Viano et al., 2009; Namjoshi et al., 2014). However, it should be noted that there are still unneglectable limitations in this scaling method due to the essential differences between human and mouse brains, such as geometry, ventricular volume and position, white/gray matter ratio, and cortical folding (Namjoshi et al., 2014; Panzer et al., 2014). According to the abovementioned scaling method, this model produced a peak linear acceleration of 12.72 g, a peak linear deceleration of 27.57 g, a peak angular acceleration of 5.19 krad/s^2^, and a peak angular deceleration of 18.38 krad/s^2^ in human head-equivalent values. The scaled peak linear parameter was lower than that for reported human brain concussion tolerances generated by the instrumented helmet data (Guskiewicz et al., 2007; Rowson et al., 2012) and below the reported linear acceleration within the range of concussion (61–144 g) (Rodowicz et al., 2015; Clark and Hoshizaki, 2016; Clark et al., 2018) but was higher than the head accelerations in volunteer soccer heading (23.5 g) (Shewchenko et al., 2005). The scaled peak angular velocity and peak angular deceleration were much higher than the reported threshold levels in the range of 3,958–12,832 rad/s^2^ that were developed from the analysis of sports collisions resulting in a human concussion (Pellman et al., 2003; Rowson et al., 2012; Rowson and Duma, 2013; Mcintosh et al., 2014; Rodowicz et al., 2015; Clark and Hoshizaki, 2016). Considering that linear kinematics predict biological outcomes better than they do angular kinematics (Namjoshi et al., 2017) and that clinical concussion may occur under many different situations, the biomechanics in our model seemed to be reasonable. However, the debate that linear or angular acceleration is a better predictor of injury severity has never been unified. In addition, there was a competitive view that the peak linear and rotational accelerations had a low correlation with injury put forward by Zhang et al. (2004) and Kleiven (2007). Moreover, another study suggested that the accelerated load curve produced by the collision rather than the peak synthetic acceleration is more representative of actual brain damage (Post et al., 2012). Therefore, further studies should focus on the dose–response relationship of rTBI, leading to biochemical and functional changes, and the relationship between combined linear and angular accelerations, such as the combined acceleration loading curve with injury severity, not just single peak value.

### Pathological Analysis

During a single injury process in this model, mouse head motion began with a transient low-amplitude acceleration and was then stopped by a short-latency high-amplitude deceleration. When rapidly shifting between acceleration and deceleration, a shear force was generated in the brains of mice due to the different densities of gray and white matter. This type of shear force resulted in axonal injury and punctate hemorrhage of microvessels in the brain parenchyma. DAI, one of the characteristic pathologies of TBI (Johnson et al., 2013), was revealed through NF-200- and β-APP-labeled immunohistochemical staining, EM, and Bielschowsky silver staining in this study. The widespread distribution of NF-200- or β-APP-labeled axonal injury verified the existence of DAI induced by this model. The outcomes, from both the microscopy of the NF-200-/β-APP-labeled axonal injury and the statistical analysis of NF-200-/β-APP-positive axon, showed that the progress of axonal injury reached its climax at 24 h post injury and was then attenuated. There was no significant difference in the number of NF-200-positive axonal varicosities between the rTBI and sham groups at 168 h post injury. However, the number of β-APP-positive axonal varicosities in the rTBI group was still significantly higher than in the sham group at 168 h post injury. The difference might be associated with the relative latency in the cleavage of β-APP (Li et al., 2010) or the accumulation of β-APP-degrading enzymes in injured axons (Johnson et al., 2010). Using EM, we observed the ultrastructural evolution of axonal injury, which ranged from cytoskeletal abnormalities, axoplasmic collapse, and swollen mitochondria to the aggregation of intra-axoplasmic electron dense material in myelinated axons of TBI mice from 24 to 168 h post injury. The cytoskeletal disruption of axons in white matter, in-axis organelle compaction, and the irregularity in axon caliber are consistent features of other rTBI models at different times following TBI (Shitaka et al., 2011; Mierzwa et al., 2015). Neurofibrillary tangles and silver-positive axonal varicosities were found in the postmortem brains of rTBI patients, while axons in the cerebral cortex of controls showed a fine linear pattern and regularity with Bielschowsky’s silver (Mckee et al., 2013) staining; thus, the disorderly distributed, swollen, and waved axons in Bielschowsky’s silver staining also verified the presence of axonal injury.

In this study, axonal injury was found in the brain stem following repetitive injury, which was consistent with evidence from humans who have experienced repeated concussive injuries. It has been reported that the atrophy of the brain stem existed in humans who suffered rTBI in the long term (Mckee et al., 2009). While, in microscopic view, rTBI exhibited the progression of its neuropathology characterized by the aggregation of abnormal proteins, like inclusions in neurofibrillary and glial tangles, these also existed in the brain stem (Mckee et al., 2009; Kiernan et al., 2015). This distinct dissemination pattern of pathological proteins was also traced in living bodies by a modern imaging technology—[^18^F]FDDNP PET brain imaging vividly showed the early axonal damage in white matter tracts and the following cumulative axonal injuries in a wide range (Barrio et al., 2015). The axonal injury in the brain stem may play a role in postural equilibrium impairment post injury.

rTBI induces immediate neuroinflammatory reactions by increasing the release of proinflammatory cytokines from activated astrocytes and microglia (Namjoshi et al., 2014). In this model, GFAP- and IBA1-positive regions were obvious and widespread in the cortex, corpus callosum, hippocampus, and brain stem, indicating extensive activation of astrocytes and microglial cells in rTBI mice. A similar phenomenon was also reported by other groups (Shitaka et al., 2011; Ekmark-Lewen et al., 2013; Chen et al., 2017), which was similar to the results of disease detection in TBI patients (Van Landeghem et al., 2007). The activation of astrocytes and microglia following TBI can either promote the recovery of acute lesions or underlie the pathobiology of memory deficits in the presence of persistent pathological stimuli (Sajja et al., 2016). Microglia associated with astrocytes and macrophages can secrete cytokines such as TNF-α and IL-1β, while the activation is initiated immediately after injury and often persists for a long time, which is related to memory deficits (Sajja et al., 2016). Accumulating evidence over the past decade indicates that the inhibition of acute cytokines and chemokine upregulation by anti-inflammatory agents improve neurological experimental TBI outcome (Yatsiv et al., 2005; Bye et al., 2007; Lloyd et al., 2008). Taken together, axon damage and dysfunction in the glial-induced changes in morphology and function may lead to impaired learning and memory in mice experiencing rTBI through this device.

### Behavioral Assessment

The righting reflex in rodents is a mesencephalic reflex that recovers function occurring earlier than thalamocortical activation during unconsciousness due to anesthesia or brain injury (Bignall, 1974). LRR in mice following TBI is considered analogous to loss of consciousness in humans after TBI, which can also function as a behavioral indicator of injury severity (Dewitt et al., 2013). An LRR of <15 min is considered mild TBI in animal TBI models, while an LRR of 15–30 min is considered moderate-severe TBI (Namjoshi et al., 2014). The average LRR duration of mice in this device after the first injury was 7.9 min, and a second TBI occurring 24 h following the first impact did not significantly extend LRR time, which indicated that mice were subjected to mild TBI in this model.

Post-concussion patients, particularly those with repetitive head impacts, often have emotional symptoms, of which depression is a major prominent symptom in humans after suffering TBI (Petraglia et al., 2012). Mice suffering rTBI in this model displayed depressive-like behavior, as manifested by the FST and TST. A similar outcome was also observed in another report, which highlighted the cumulative effect of rTBI (Petraglia et al., 2014). Cognitive dysfunction is one of the post-concussion symptoms of rTBI, including impaired executive function; learning, memory, and attention disorders; and decreased speed of processing tasks (Pavlovic et al., 2019). Many studies implied that rTBI was associated with prolonged cognitive impairment (Collins et al., 1999; Guskiewicz et al., 2005; Preiss-Farzanegan et al., 2009). However, there have been other contradictory findings (Broglio et al., 2006; Collie et al., 2006; Bruce and Echemendia, 2009). Although controversy exists, the rTBI mice in this model manifested obvious spatial learning and memory deficits during the acquisition trials of the MWM task at postinjury acute and subacute phases and at 1 and 3 months. Similar changes were also reported by other groups (Shitaka et al., 2011; Mouzon et al., 2012; Namjoshi et al., 2014; Petraglia et al., 2014). Chen et al. (2017) also observed prolonged spatial learning and memory defects at 6 months in mice after injury with three impacts at 24-h intervals, which suggested that a wider range of time after injury may be needed for further research.

In summary, we reported a new mouse model of rTBI triggered by combined linear and angular accelerations, which effectively replicated the clinical injury process. The allowance of biomechanical measurements showed that head kinematic parameters in this model were comparable to those in humans. The following evaluation of this model, through histopathological analyses and behavioral assessments, indicated that our results were comparable to clinical observations in humans and consistent with other rodent models of rTBI. All these integrations, plus with its innate surgical-free and highly reproducible characteristics, make it a suitable mouse model for rTBI research. Future studies will further investigate the relationship between biomechanics and corresponding behavioral and neuropathological findings.

## Data Availability Statement

All datasets generated for this study are included in the article/supplementary material.

## Ethics Statement

The animal study was reviewed and approved by The Animal Care Committee of the Shanghai Jiao Tong University School of Medicine.

## Author Contributions

D-FF conceived the study and promoted the progress of the project. KC performed the experiments of histology, morphology, and biochemistry experiments. HG performed the experiments of animal behavior. LZ participated in analysis and interpretation of the data and critically revised the manuscript. KC, HG, and D-FF designed the experiments and wrote the manuscript. All authors agree that all issues related to the accuracy or completeness of the manuscript have been properly investigated and resolved, and the version to be released is finally approved.

## Conflict of Interest

The authors declare that the research was conducted in the absence of any commercial or financial relationships that could be construed as a potential conflict of interest.

## References

[B1] Adelson P. D. Jenkins L. W. Hamilton R. L. Robichaud P. Tran M. P. Kochanek P. M. (2001). Histopathologic response of the immature rat to diffuse traumatic brain injury. *J. Neurotrauma* 18 967–976. 10.1089/08977150152693674 11686497

[B2] Barrio J. R. Small G. W. Wong K. P. Huang S. C. Liu J. Merrill D. A. (2015). In vivo characterization of chronic traumatic encephalopathy using [F-18]FDDNP PET brain imaging. *Proc. Natl. Acad. Sci. U.S.A.* 112 E2039–E2047. 25848027 10.1073/pnas.1409952112 PMC4413350

[B3] Bignall K. E. (1974). Ontogeny of levels of neural organization: the righting reflex as a model. *Exp. Neurol.* 42 566–573. 10.1016/0014-4886(74)90079-x 4828676

[B4] Bolton A. N. Saatman K. E. (2014). Regional neurodegeneration and gliosis are amplified by mild traumatic brain injury repeated at 24-hour intervals. *J. Neuropathol. Exp. Neurol.* 73 933–947. 10.1097/NEN.0000000000000115 25232942 PMC4170569

[B5] Broglio S. P. Ferrara M. S. Piland S. G. Anderson R. B. Collie A. (2006). Concussion history is not a predictor of computerised neurocognitive performance. *Br. J. Sports Med.* 40 802–805; discussion 802–805. 10.1136/bjsm.2006.028019 16929049 PMC2564398

[B6] Bruce J. M. Echemendia R. J. (2009). History of multiple self-reported concussions is not associated with reduced cognitive abilities. *Neurosurgery* 64 100–106;discussion 106. 10.1227/01.NEU.0000336310.47513.C8 19145158

[B7] Bye N. Habgood M. D. Callaway J. K. Malakooti N. Potter A. Kossmann T. (2007). Transient neuroprotection by minocycline following traumatic brain injury is associated with attenuated microglial activation but no changes in cell apoptosis or neutrophil infiltration. *Exp. Neurol.* 204 220–233. 10.1016/j.expneurol.2006.10.013 17188268

[B8] Cernak I. Savic J. Malicevic Z. Zunic G. Radosevic P. Ivanovic I. (1996). Involvement of the central nervous system in the general response to pulmonary blast injury. *J. Trauma* 40 S100–S104. 8606388 10.1097/00005373-199603001-00023

[B9] Changa A. R. Vietrogoski R. A. Carmel P. W. (2018). Dr Harrison Martland and the history of punch drunk syndrome. *Brain* 141 318–321. 10.1093/brain/awx349 29325051

[B10] Chen H. Desai A. Kim H. Y. (2017). Repetitive closed-head impact model of engineered rotational acceleration induces long-term cognitive impairments with persistent astrogliosis and microgliosis in mice. *J. Neurotrauma* 34 2291–2302. 10.1089/neu.2016.4870 28288551 PMC5510798

[B11] Clark J. M. Hoshizaki T. B. (2016). the ability of men’s lacrosse helmets to reduce the dynamic impact response for different striking techniques in women’s field lacrosse. *Am. J. Sports Med.* 44 1047–1055. 10.1177/0363546515623272 26831628

[B12] Clark J. M. Hoshizaki T. B. Gilchrist M. D. (2018). Assessing women’s lacrosse head impacts using finite element modelling. *J. Mech. Behav. Biomed. Mater.* 80 20–26. 10.1016/j.jmbbm.2018.01.020 29414471

[B13] Collie A. Mccrory P. Makdissi M. (2006). Does history of concussion affect current cognitive status? *Br. J. Sports Med.* 40 550–551. 10.1136/bjsm.2005.019802 16720889 PMC2465097

[B14] Collins M. W. Grindel S. H. Lovell M. R. Dede D. E. Moser D. J. Phalin B. R. (1999). Relationship between concussion and neuropsychological performance in college football players. *JAMA* 282 964–970. 10485682 10.1001/jama.282.10.964

[B15] Corso P. Finkelstein E. Miller T. Fiebelkorn I. Zaloshnja E. (2006). Incidence and lifetime costs of injuries in the United States. *Inj. Prev.* 12 212–218. 10.1136/ip.2005.010983 16887941 PMC2586784

[B16] Dewitt D. S. Perez-Polo R. Hulsebosch C. E. Dash P. K. Robertson C. S. (2013). Challenges in the development of rodent models of mild traumatic brain injury. *J. Neurotrauma* 30 688–701. 10.1089/neu.2012.2349 23286417

[B17] Dixon C. E. Clifton G. L. Lighthall J. W. Yaghmai A. A. Hayes R. L. (1991). A controlled cortical impact model of traumatic brain injury in the rat. *J. Neurosci. Methods* 39 253–262. 1787745 10.1016/0165-0270(91)90104-8

[B18] Dixon C. E. Lyeth B. G. Povlishock J. T. Findling R. L. Hamm R. J. Marmarou A. (1987). A fluid percussion model of experimental brain injury in the rat. *J. Neurosurg.* 67 110–119. 3598659 10.3171/jns.1987.67.1.0110

[B19] Ekmark-Lewen S. Flygt J. Kiwanuka O. Meyerson B. J. Lewen A. Hillered L. (2013). Traumatic axonal injury in the mouse is accompanied by a dynamic inflammatory response, astroglial reactivity and complex behavioral changes. *J. Neuroinflammation* 10:44. 10.1186/1742-2094-10-44 23557178 PMC3651302

[B20] Geddes J. F. Vowles G. H. Nicoll J. A. Revesz T. (1999). Neuronal cytoskeletal changes are an early consequence of repetitive head injury. *Acta Neuropathol.* 98 171–178. 10.1007/s004010051066 10442557

[B21] Goldstein L. E. Fisher A. M. Tagge C. A. Zhang X. L. Velisek L. Sullivan J. A. (2012). Chronic traumatic encephalopathy in blast-exposed military veterans and a blast neurotrauma mouse model. *Sci. Transl. Med.* 4 134ra160. 10.1126/scitranslmed.3003716 PMC3739428 22593173

[B22] Greenwald R. M. Gwin J. T. Chu J. J. Crisco J. J. (2008). Head impact severity measures for evaluating mild traumatic brain injury risk exposure. *Neurosurgery* 62 789–798; discussion 798. 10.1227/01.neu.0000318162.67472.ad 18496184 PMC2790598

[B23] Guskiewicz K. M. Marshall S. W. Bailes J. Mccrea M. Cantu R. C. Randolph C. (2005). Association between recurrent concussion and late-life cognitive impairment in retired professional football players. *Neurosurgery* 57 719–726; discussion 719–726. 10.1227/01.neu.0000175725.75780.dd 16239884

[B24] Guskiewicz K. M. Mccrea M. Marshall S. W. Cantu R. C. Randolph C. Barr W. (2003). Cumulative effects associated with recurrent concussion in collegiate football players: the NCAA concussion study. *JAMA* 290 2549–2555. 14625331 10.1001/jama.290.19.2549

[B25] Guskiewicz K. M. Mihalik J. P. Shankar V. Marshall S. W. Crowell D. H. Oliaro S. M. (2007). Measurement of head impacts in collegiate football players: relationship between head impact biomechanics and acute clinical outcome after concussion. *Neurosurgery* 61 1244–1252; discussion 1252–1243. 18162904 10.1227/01.neu.0000306103.68635.1a

[B26] Gutierrez E. Huang Y. Haglid K. Bao F. Hansson H. A. Hamberger A. (2001). A new model for diffuse brain injury by rotational acceleration: I model, gross appearance, and astrocytosis. *J. Neurotrauma* 18 247–257. 10.1089/08977150151070874 11284546

[B27] Johnson V. E. Stewart W. Smith D. H. (2010). Traumatic brain injury and amyloid-beta pathology: a link to Alzheimer’s disease? *Nat. Rev. Neurosci.* 11 361–370. 10.1038/nrn2808 20216546 PMC3979339

[B28] Johnson V. E. Stewart W. Smith D. H. (2013). Axonal pathology in traumatic brain injury. *Exp. Neurol.* 246 35–43. 10.1016/j.expneurol.2012.01.013 22285252 PMC3979341

[B29] Karton C. Rousseau P. Vassilyadi M. Hoshizaki T. B. (2014). The evaluation of speed skating helmet performance through peak linear and rotational accelerations. *Br. J. Sports Med.* 48 46–50. 10.1136/bjsports-2012-091583 23314891

[B30] Kiernan P. T. Montenigro P. H. Solomon T. M. Mckee A. C. (2015). Chronic traumatic encephalopathy: a neurodegenerative consequence of repetitive traumatic brain injury. *Semin. Neurol.* 35 20–28. 10.1055/s-0035-1545080 25714864

[B31] Kleiven S. (2007). Predictors for traumatic brain injuries evaluated through accident reconstructions. *Stapp Car Crash J.* 51 81–114. 18278592 10.4271/2007-22-0003

[B32] Langburt W. Cohen B. Akhthar N. O’neill K. Lee J. C. (2001). Incidence of concussion in high school football players of Ohio and Pennsylvania. *J. Child Neurol.* 16 83–85. 10.1177/088307380101600203 11292230

[B33] Lasry O. Liu E. Y. Powell G. A. Ruel-Laliberte J. Marcoux J. Buckeridge D. L. (2017). Epidemiology of recurrent traumatic brain injury in the general population: a systematic review. *Neurology* 89 2198–2209. 10.1212/WNL.0000000000004671 29070664 PMC5696641

[B34] Li X. Y. Li J. Feng D. F. Gu L. (2010). Diffuse axonal injury induced by simultaneous moderate linear and angular head accelerations in rats. *Neuroscience* 169 357–369. 10.1016/j.neuroscience.2010.04.075 20451584

[B35] Lighthall J. W. (1988). Controlled cortical impact: a new experimental brain injury model. *J. Neurotrauma* 5 1–15. 10.1089/neu.1988.5.1 3193461

[B36] Lloyd E. Somera-Molina K. Van Eldik L. J. Watterson D. M. Wainwright M. S. (2008). Suppression of acute proinflammatory cytokine and chemokine upregulation by post-injury administration of a novel small molecule improves long-term neurologic outcome in a mouse model of traumatic brain injury. *J. Neuroinflammation* 5:28. 10.1186/1742-2094-5-28 18590543 PMC2483713

[B37] Longhi L. Saatman K. E. Fujimoto S. Raghupathi R. Meaney D. F. Davis J. (2005). Temporal window of vulnerability to repetitive experimental concussive brain injury. *Neurosurgery* 56 364–374; discussion 364–374. 10.1227/01.neu.0000149008.73513.44 15670384

[B38] Marmarou A. Foda M. A. Van Den Brink W. Campbell J. Kita H. Demetriadou K. (1994). A new model of diffuse brain injury in rats. Part I: pathophysiology and biomechanics. *J. Neurosurg.* 80 291–300. 10.3171/jns.1994.80.2.0291 8283269

[B39] Mcintosh A. S. Patton D. A. Frechede B. Pierre P. A. Ferry E. Barthels T. (2014). The biomechanics of concussion in unhelmeted football players in Australia: a case-control study. *BMJ Open* 4:e005078. 10.1136/bmjopen-2014-005078 24844272 PMC4039841

[B40] Mcintosh T. K. Vink R. Noble L. Yamakami I. Fernyak S. Soares H. (1989). Traumatic brain injury in the rat: characterization of a lateral fluid-percussion model. *Neuroscience* 28 233–244. 10.1016/0306-4522(89)90247-9 2761692

[B41] Mckee A. C. Cantu R. C. Nowinski C. J. Hedley-Whyte E. T. Gavett B. E. Budson A. E. (2009). Chronic traumatic encephalopathy in athletes: progressive tauopathy after repetitive head injury. *J. Neuropathol. Exp. Neurol.* 68 709–735. 10.1097/NEN.0b013e3181a9d503 19535999 PMC2945234

[B42] Mckee A. C. Stern R. A. Nowinski C. J. Stein T. D. Alvarez V. E. Daneshvar D. H. (2013). The spectrum of disease in chronic traumatic encephalopathy. *Brain* 136 43–64. 10.1093/brain/aws307 23208308 PMC3624697

[B43] Meaney D. F. Smith D. H. (2011). Biomechanics of concussion. *Clin. Sports Med.* 30 19–31, vii. 21074079 10.1016/j.csm.2010.08.009 PMC3979340

[B44] Mierzwa A. J. Marion C. M. Sullivan G. M. Mcdaniel D. P. Armstrong R. C. (2015). Components of myelin damage and repair in the progression of white matter pathology after mild traumatic brain injury. *J. Neuropathol. Exp. Neurol.* 74 218–232. 10.1097/NEN.0000000000000165 25668562 PMC4327393

[B45] Mouzon B. Chaytow H. Crynen G. Bachmeier C. Stewart J. Mullan M. (2012). Repetitive mild traumatic brain injury in a mouse model produces learning and memory deficits accompanied by histological changes. *J. Neurotrauma* 29 2761–2773. 10.1089/neu.2012.2498 22900595 PMC13588329

[B46] Namjoshi D. R. Cheng W. H. Bashir A. Wilkinson A. Stukas S. Martens K. M. (2017). Defining the biomechanical and biological threshold of murine mild traumatic brain injury using CHIMERA (Closed Head Impact Model of Engineered Rotational Acceleration). *Exp. Neurol.* 292 80–91. 10.1016/j.expneurol.2017.03.003 28274861

[B47] Namjoshi D. R. Cheng W. H. Mcinnes K. A. Martens K. M. Carr M. Wilkinson A. (2014). Merging pathology with biomechanics using CHIMERA (Closed-Head Impact Model of Engineered Rotational Acceleration): a novel, surgery-free model of traumatic brain injury. *Mol. Neurodegener.* 9:55. 10.1186/1750-1326-9-55 25443413 PMC4269957

[B48] Omalu B. Bailes J. Hamilton R. L. Kamboh M. I. Hammers J. Case M. (2011). Emerging histomorphologic phenotypes of chronic traumatic encephalopathy in American athletes. *Neurosurgery* 69 173–183; discussion 183. 10.1227/NEU.0b013e318212bc7b 21358359

[B49] Panzer M. B. Wood G. W. Bass C. R. (2014). Scaling in neurotrauma: how do we apply animal experiments to people? *Exp. Neurol.* 261 120–126. 10.1016/j.expneurol.2014.07.002 25035134

[B50] Pavlovic D. Pekic S. Stojanovic M. Popovic V. (2019). Traumatic brain injury: neuropathological, neurocognitive and neurobehavioral sequelae. *Pituitary* 22 270–282. 10.1007/s11102-019-00957-9 30929221

[B51] Pellman E. J. Viano D. C. Tucker A. M. Casson I. R. Waeckerle J. F. (2003). Concussion in professional football: reconstruction of game impacts and injuries. *Neurosurgery* 53 799–812; discussion 812–794. 14519212 10.1093/neurosurgery/53.3.799

[B52] Petraglia A. L. Maroon J. C. Bailes J. E. (2012). From the field of play to the field of combat: a review of the pharmacological management of concussion. *Neurosurgery* 70 1520–1533; discussion 1533. 10.1227/NEU.0b013e31824cebe8 22289786

[B53] Petraglia A. L. Plog B. A. Dayawansa S. Chen M. Dashnaw M. L. Czerniecka K. (2014). The spectrum of neurobehavioral sequelae after repetitive mild traumatic brain injury: a novel mouse model of chronic traumatic encephalopathy. *J. Neurotrauma* 31 1211–1224. 10.1089/neu.2013.3255 24766454 PMC4082360

[B54] Porsolt R. D. Bertin A. Jalfre M. (1977). Behavioral despair in mice: a primary screening test for antidepressants. *Arch. Int. Pharmacodyn. Ther.* 229 327–336. 596982

[B55] Post A. Hoshizaki B. Gilchrist M. D. (2012). Finite element analysis of the effect of loading curve shape on brain injury predictors. *J. Biomech.* 45 679–683. 10.1016/j.jbiomech.2011.12.005 22239921

[B56] Preiss-Farzanegan S. J. Chapman B. Wong T. M. Wu J. Bazarian J. J. (2009). The relationship between gender and postconcussion symptoms after sport-related mild traumatic brain injury. *PM R* 1 245–253. 10.1016/j.pmrj.2009.01.011 19627902 PMC5237580

[B57] Rodowicz K. A. Olberding J. E. Rau A. C. (2015). Head injury potential and the effectiveness of headgear in women’s lacrosse. *Ann. Biomed. Eng.* 43 949–957. 10.1007/s10439-014-1154-x 25326438

[B58] Rowson S. Duma S. M. (2013). Brain injury prediction: assessing the combined probability of concussion using linear and rotational head acceleration. *Ann. Biomed. Eng.* 41 873–882. 10.1007/s10439-012-0731-0 23299827 PMC3624001

[B59] Rowson S. Duma S. M. Beckwith J. G. Chu J. J. Greenwald R. M. Crisco J. J. (2012). Rotational head kinematics in football impacts: an injury risk function for concussion. *Ann. Biomed. Eng.* 40 1–13. 10.1007/s10439-011-0392-4 22012081 PMC10465647

[B60] Sajja V. S. Hlavac N. Vandevord P. J. (2016). Role of glia in memory deficits following traumatic brain injury: biomarkers of glia dysfunction. *Front. Integr. Neurosci.* 10:7. 10.3389/fnint.2016.00007 26973475 PMC4770450

[B61] Sauerbeck A. D. Fanizzi C. Kim J. H. Gangolli M. Bayly P. V. Wellington C. L. (2018). modCHIMERA: a novel murine closed-head model of moderate traumatic brain injury. *Sci. Rep.* 8:7677. 10.1038/s41598-018-25737-6 29769541 PMC5955903

[B62] Shewchenko N. Withnall C. Keown M. Gittens R. Dvorak J. (2005). Heading in football. Part 1: development of biomechanical methods to investigate head response. *Br. J. Sports Med.* 39(Suppl. 1), i10–i25. 10.1136/bjsm.2005.019034 16046351 PMC1765311

[B63] Shitaka Y. Tran H. T. Bennett R. E. Sanchez L. Levy M. A. Dikranian K. (2011). Repetitive closed-skull traumatic brain injury in mice causes persistent multifocal axonal injury and microglial reactivity. *J. Neuropathol. Exp. Neurol.* 70 551–567. 10.1097/NEN.0b013e31821f891f 21666502 PMC3118973

[B64] Steru L. Chermat R. Thierry B. Simon P. (1985). The tail suspension test: a new method for screening antidepressants in mice. *Psychopharmacology (Berl)* 85 367–370. 10.1007/bf00428203 3923523

[B65] Van Landeghem F. K. Weiss T. Oehmichen M. Von Deimling A. (2007). Cellular localization of pituitary adenylate cyclase-activating peptide (PACAP) following traumatic brain injury in humans. *Acta Neuropathol.* 113 683–693. 10.1007/s00401-007-0208-7 17431645

[B66] Viano D. C. Hamberger A. Bolouri H. Saljo A. (2009). Concussion in professional football: animal model of brain injury–part 15. *Neurosurgery* 64 1162–1173; discussion 1173. 10.1227/01.NEU.0000345863.99099.C7 19487897

[B67] Xiao-Sheng H. Sheng-Yu Y. Xiang Z. Zhou F. Jian-Ning Z. (2000). Diffuse axonal injury due to lateral head rotation in a rat model. *J. Neurosurg.* 93 626–633. 10.3171/jns.2000.93.4.0626 11014541

[B68] Xiong Y. Mahmood A. Chopp M. (2013). Animal models of traumatic brain injury. *Nat. Rev. Neurosci.* 14 128–142. 10.1038/nrn3407 23329160 PMC3951995

[B69] Yatsiv I. Grigoriadis N. Simeonidou C. Stahel P. F. Schmidt O. I. Alexandrovitch A. G. (2005). Erythropoietin is neuroprotective, improves functional recovery, and reduces neuronal apoptosis and inflammation in a rodent model of experimental closed head injury. *FASEB J.* 19 1701–1703. 10.1096/fj.05-3907fje 16099948

[B70] Zhang L. Yang K. H. King A. I. (2004). A proposed injury threshold for mild traumatic brain injury. *J. Biomech. Eng.* 126 226–236. 10.1115/1.1691446 15179853

